# Association between cagemate number and risk of death in mice: a time-varying covariate analysis using Cox frailty models

**DOI:** 10.1007/s11357-025-02080-z

**Published:** 2026-01-09

**Authors:** Thirupathi R. Mokalla, Erik S. Parker, Daniel L. Smith, Luis-Enrique Becerra-Garcia, Olivia C. Robertson, Deependra K. Thapa, Mounika Kandukuri, Bret M. Rust, David B. Allison

**Affiliations:** 1 Department of Pediatrics and USDA Children’s Nutrition Research Center, Baylor College of Medicine, Houston, TX USA; 2 Department of Epidemiology and Biostatistics, Indiana University School of Public Health, Bloomington, IN USA; 3 Department of Nutrition Sciences, University of Alabama at Birmingham, Birmingham, AL USA; 4 Department of Applied Health Science, Indiana University School of Public Health, Bloomington, IN USA

**Keywords:** Social housing, Interventions testing program, Cox frailty models

## Abstract

Social housing is desirable for the health and well-being of laboratory mice, as social interactions with conspecifics influence both behavioral and physiological outcomes. Although group housing benefits social species, it can introduce variability in mortality outcomes, and raise welfare concerns, particularly with the emergence of aggression or fluctuating cage densities. Despite this, few studies have evaluated how changes in the number of living cagemates over time are associated with survival, particularly in a sex-specific manner. We analyzed data from the National Institute on Aging’s Interventions Testing Program (ITP; *n* = 2635 UM-HET3 mice), across three research sites to assess whether housing density influenced longevity differently in male and female mice. Mice were housed in same-sex cages (median = 3 per cage) without reassignment after cagemate death. We applied Cox frailty models incorporating nested random effects for cage and site, with fixed effects for sex, treatment, and time-varying number of living cagemates to estimate hazard ratios, which allowed us to assess the instantaneous risk of death associated with changes in cagemate number. Results showed a significant main effect of the number of living cagemates on mortality and a significant interaction between sex and cagemate count, indicating sex-specific responses. Female mice exhibited a pronounced increase in mortality rate as cage density declined, suggesting a potential role of social buffering in longevity. These findings emphasize the importance of considering social housing dynamics, particularly for female mice, in both experimental design and animal welfare protocols.

**Graphical Abstract:**

**Figure:**
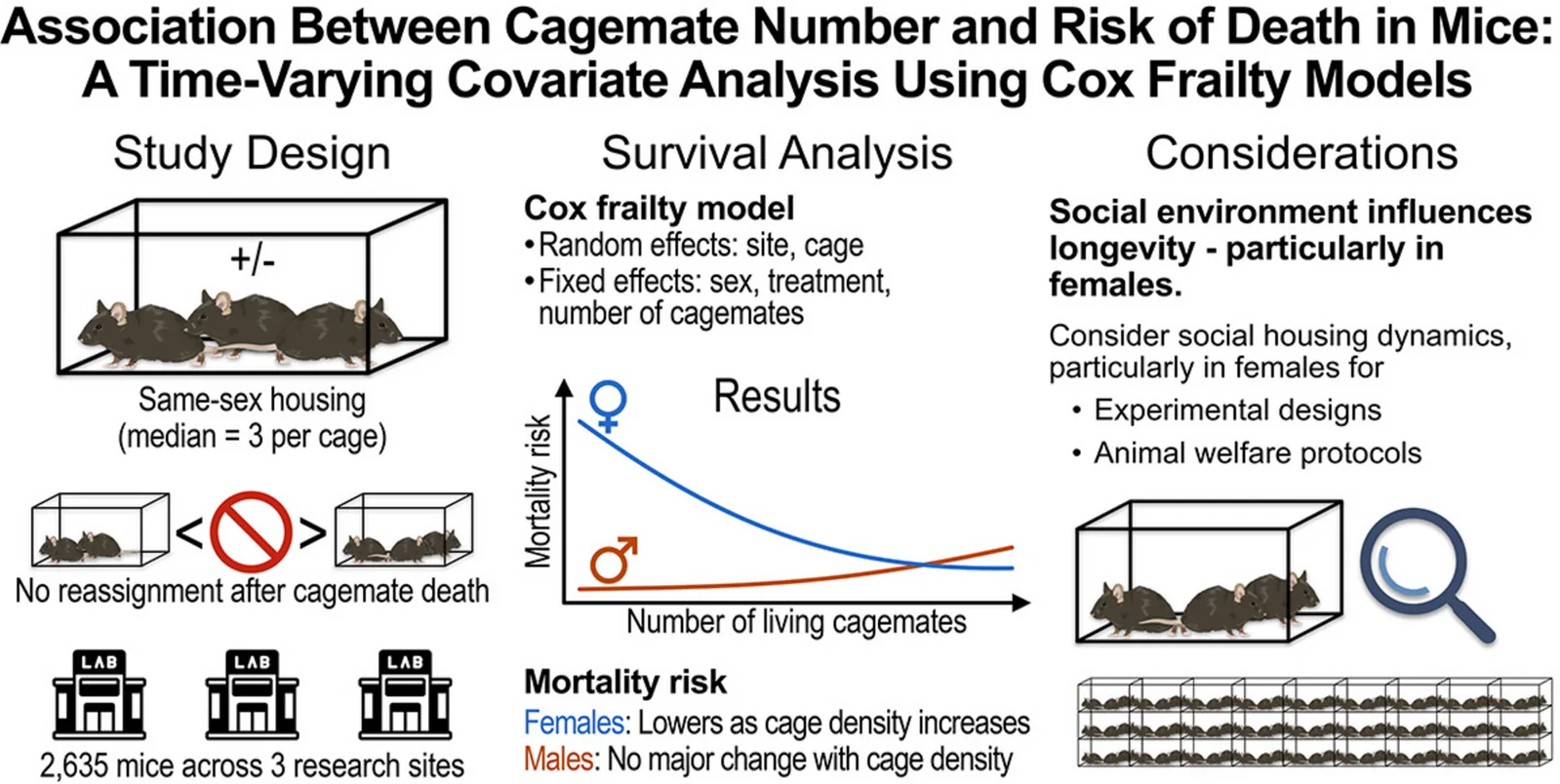

## Introduction

Guidelines for housing mice in scientific research emphasize providing appropriate social contact to ensure normal development and well-being [1]. Housing density, the number of animals in a cage relative to a two- or three-dimensional space metric, is sometimes used interchangeably with the number of animals per cage, but cage sizes differ among research sites such that housing density may differ even if the number of animals per cage does not [2, 3]. Co-housing enables animals to engage in species-specific social behavior, and social support improves physiological outcomes and ergo enhances model quality [4, 5]. Maintaining proper social habitation is a concern for social animals as individual housing stresses animals, especially female mice [1, 6, 7]. In males, social housing can give rise to aggression and fighting, leading to pain, injury, and even death [8]. Furthermore, studies have shown that variability in cage density is associated with changes in the behavior and physiology of the animals, thereby influencing a study’s outcomes and conclusions [2, 9].

Social interactions play a crucial role in shaping health outcomes and mortality rates in rodent models. The dynamics of social dominance hierarchies in group-housed conditions versus isolation are significantly associated with physiological and behavioral responses in rodents [10, 11]. Female mice exhibit social behaviors, including increased “cuddling” or “huddling” interactions. Thus, female mice seem to be particularly affected by the presence of other cagemates [12]. Social behavior differences between sexes may be a relevant consideration in long-term study design.

Yet, intra-cage housing density in longevity analyses is seldom studied, nor have changes in housing density over time due to natural mortality or sacrifice been considered [13, 14]. To address this gap in the literature, we investigated both general and sex-specific associations between changing cage densities and the hazard of death using Cox frailty models with time-varying covariates. We initially set out to evaluate the reproducibility and robustness of findings from a previously published Interventions Testing Program (ITP) cages study by re-analyzing the data with improved statistical methods [15]. During this re-analysis, we identified some possible sex-specific associations between changing cage densities and mouse survival. Although these association were not hypothesized a priori, their emergence highlights the importance of considering sex-specific social dynamics in animal housing design. The present study therefore illustrates how reproducibility efforts can uncover hidden sources of variability and lead to novel insights in preclinical research, and we wish to be transparent that they were not part of the original study aims and should be interpreted in light of their exploratory origin.

## Methods

The data used for the analyses presented in this paper were obtained from the ITP and were described previously in Harrison et al.,2023 [13] and Parker et al., 2025 [15]. Briefly, 2,635 UM-HET3 mice were housed across three sites (The Jackson Laboratory, the University of Michigan, and the University of Texas), with an average of roughly 875 mice per location. At each location, mice were weaned at 42 days of age and assigned to sex-specific cages, with a median of 3, a mean of 3.39, and a maximum of 4 mice per cage at the start of the study period. These cages were then block randomized to a treatment group. Through the course of the study, animals were not rehoused to new cages when their original cagemates died such that their cage assigned at the start of the study period was their cage until death or study end.

To evaluate how co-housing is associated with differences in the hazard of death in male and female mice, we constructed a Cox frailty model with nested frailty terms for cage within research site. Aside from the frailty terms, this model also included fixed effects for animal sex, treatment group (drug given), an interaction between sex and treatment group, and a time-varying covariate capturing the number of cagemates alive at any given point in time. In these models, the outcome modeled was the hazard of death, which represents the instantaneous risk of death at a given time, conditional on survival up to that time. This differs from survival probability (the chance of being alive beyond a given time) and from mortality proportion (the fraction of animals that die during a period). Type 3 sum of squares were used to assess the significance of the two-way interaction term (sex by treatment) and main effects of sex and treatment. Further, pairwise estimated marginal means comparing each treatment to the control within each sex were used to determine whether there were statistically significant differences in the hazard of death between treatment groups, by sex.

We next explicitly tested the hypothesis that the longevity of male and female mice would be differentially affected by the number of living cagemates at any given point in time. To assess this, we fit a Cox frailty model similar to the one used to examine our first hypothesis, this time with an additional two-way interaction between sex and the time-varying covariate for number of living cagemates at any given time. As before, the statistical significance of the two-way interaction between sex and number of cagemates alive was assessed using type 3 sum of squares, with the two-way interaction between treatment and sex and the main effects. Finally, pairwise estimated marginal means were compared to determine whether there were statistically significant differences between sexes in the hazard of death when a given animal was surrounded by a different number of living cagemates.

All statistical analyses were performed in R (version 4.3.0) using the survival (version 3.5–5) [16], coxme (version 2.2–22) [17], and emmeans (version 1.8.8) [18] packages.

## Results

Our first Cox frailty model, including the main effects of treatment, sex, number of cagemates, as well as the treatment by sex interaction, demonstrated a significant association between the number of living cagemates and the hazard of death in these mice, independent of sex or treatment group (p < 0.001; Table 1).

**Table 1:** Type 3 sum of squares results for Cox frailty models including time-varying covariates for number of cagemates alive at any given time

*Variable*	Main effects model	Cagemate number X Sex interaction model
	*p-value*	*p-value*
Treatment	0.272	0.418
Sex	0.942	** < 0.001**
Number of living cagemates	** < 0.001**	** < 0.001**
Treatment X Sex	0.139	0.141
Number of living cagemates X Sex	-	** < 0.001**

**Fig. 1:**
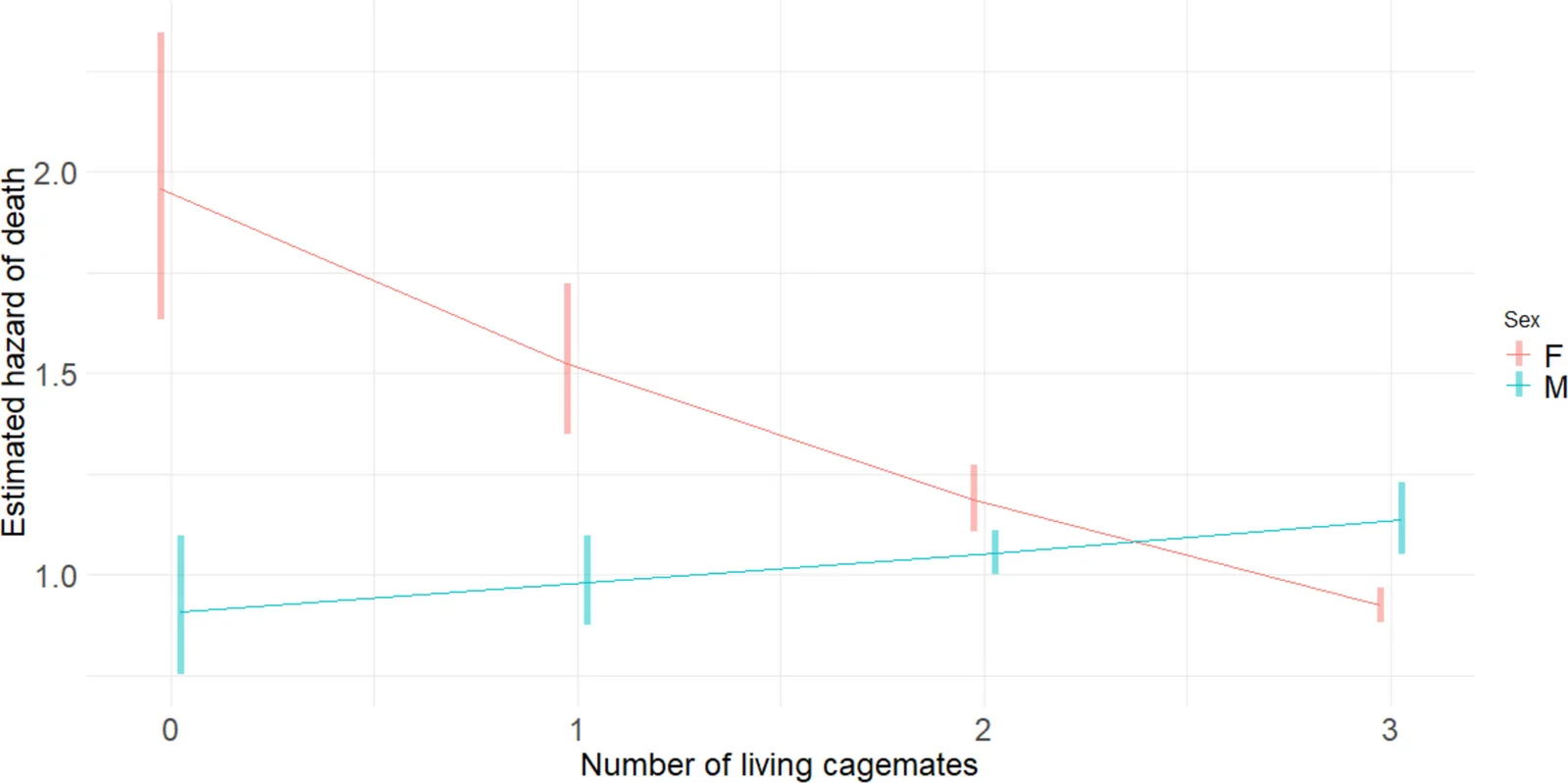
Estimated hazards of death for a male or female animal with the given number of living cagemates

The relationship between housing density and survival was sex-specific. In our second Cox frailty model, we included the same variables as in our first model, with the addition of an interaction between number of living cagemates at a given time and sex. From this model we saw that there was a statistically significant interaction between the number of living cagemates and sex (p < 0.001; Table 1). This interaction indicates that male and female mice have different predicted hazards of death as the number of living cagemates decreases over time. When we examined the estimated hazard of death as a function of the number of surviving cagemates within each sex, we saw that female mice had a pronounced increase in the hazard of death as the number of living cagemates decreased (Table 2; Fig. 1). However, in male mice, hazard of death was not related to the number of living cagemates but, in fact, decreased non-significantly over time.

**Table 2:** Estimated hazards of death for a male or female animal with the given number of living cagemates

Number of living cagemates	*Estimated death hazard*	*SE*
Female mice		
Three	0.92	0.02
Two	1.19	0.04
One	1.52	0.1
Zero (only one animal remains alive in cage)	1.96	0.2
Male mice		
Three	1.14	0.05
Two	1.06	0.03
One	0.98	0.06
Zero	0.91	0.09

## Discussion

Housing conditions represent an important environmental variable playing a critical role in the assessment of mouse behavior and physiology [19, 20]. However, less attention has focused on how changes in housing density affect primary study outcomes.

The social conditions for housing mice potentially affect study outcomes yet are rarely considered and are inadequately standardized, highlighting a significant gap in current research practices and reporting.

While innate fear behavior remains consistent throughout the lifespan in both sexes, studies have aimed to uncover subtle differences in coping mechanisms that may influence how male and female mice respond to external threats [8, 21, 22]. Crucially, understanding differences in coping mechanisms between sexes can provide insight into how environmental factors may be associated with the differential stress responses of male and female mice. In this study, we investigated the association between the number of living cagemates and survival in both male and female mice. Our main aim was to determine whether the number of cagemates has a significant influence on mortality rates in male and female mice. Additionally, we assessed the potential association of time-varying cagemate number on survival within each sex.

## Study findings and interpretation

Our analysis revealed striking differences between sexes in the association between the number of cagemates in a cage and mortality rate. Female mice demonstrated a pronounced vulnerability to social isolation, with hazard of death increasing dramatically from 0.92 when housed with three cagemates to 1.96 when alone in the cage.

These findings align with current evidence on sex-specific social tendencies in mice. Female rodents display increased sociability in group housing contexts and may benefit physiologically from opportunities for social interaction, including nesting proximity and potential cooperative care [6, 23]. The increased mortality rate observed in isolated females may reflect the stress from social deprivation, which can alter immune function, stress hormone levels, and overall physiological well-being [24]. In previous research, individual housing of female mice altered postsurgical behavior and impaired recovery [6], supporting our observation that female mice are particularly vulnerable to the absence of social companions.

The contrasting lack of association in male mice may reflect the different social dynamics typical of male mouse behavior. Male mice often establish dominance hierarchies that can lead to aggressive interactions and stress [8, 25]. Our data demonstrate an intriguing contrast in the social dynamics of male and female mice where any positive association between social companionship and mortality rate in male mice is outweighed by the stress of social competition and the potential of aggression. This interpretation is consistent with studies indicating that male mice can experience substantial stress in group housing situations, particularly when social hierarchies are disrupted [8].

## Comparison with existing literature

Our results provide novel quantitative evidence for sex-specific responses to social housing that complement existing qualitative observations. Previously, singly and group-housed mice were compared, but the relationship between the number of living cagemates and mortality on surviving animals was not assessed. However, the study by Ikeno et al. [26], which examined calorie-restricted male B6 mice, reported no difference in lifespan of male mice housed singly or in groups of four. This study finding is consistent with our observations in male mice. Other studies have examined housing density or social environment in relation to physiological or welfare outcomes, but without evaluating how dynamic shifts in cage density influence mortality [2, 14, 26–28]. Moreover, whereas previous studies have noted that female rats appear more affected by number of cagemates [23] and that individual housing can be particularly stressful for females [6, 7], our study is among the first to demonstrate this association using survival analysis with time-varying covariates that account for the dynamic nature of cagemate living over time.

The magnitude of the association we observed in females, with mortality rate more than doubling when transitioning from group to individual housing, suggests that the social environment may be as influential as many pharmacological interventions in determining survival outcomes. This finding has important implications for the interpretation of longevity studies, as housing conditions may introduce bias if not adequately controlled for in study design or analysis.

## Limitations

Our analysis was conducted on data from a single longevity study [13]; replication in other cohorts would strengthen these findings. We were unable to account for other potentially important factors such as dominance status within cages, specific social behaviors, or environmental enrichment that might modify the observed associations with the number of cagemates alive. Additionally, the original study was designed to test pharmacological interventions rather than housing-related associations, so some relevant behavioral or physiological measures were not collected. Also, lacking cause-of-death data prevents us from determining with absolute certainty whether survival differences can be attributed to social factors, underlying health conditions, or disease outbreaks in the crates. Without this, some mortality clusters may reflect contagion rather than purely social effects. This study was conducted in UM-HET3 mice, which exhibit strain- and sex-specific social behaviors, including male aggression, that may influence housing dynamics and mortality outcomes. Therefore, our findings may not be generalizable to other mouse strains or species (e.g., rats), particularly those with lower male aggression. Future studies across multiple genetic backgrounds and species will be important to determine the broader applicability of these results.

## Implications for laboratory practice

These findings have practical implications for laboratory mouse husbandry and experimental design. First, maintaining consistent group sizes throughout longevity studies may be important for controlling potential confounding variables. Second, the sex-specific nature of these associations indicates that housing protocols may need to be tailored differently for male and female mice. For studies involving female mice, particular attention should be paid to minimizing social isolation, whereas while for male mice, individual housing may be acceptable or even preferable in some circumstances.

The progressive loss of cagemates in aging studies, as animals die over time and survivors become increasingly isolated, represents an underrecognized confounding factor that may influence study outcomes. Future longevity research in mice should consider changes in cagemate number resulting from death and loss of cagemates in mouse models using long-term interventions to assess physiologic outcomes not restricted to longevity. The impact of rehousing surviving mice after deaths to maintain cage mate number has not been studied. In female mice, such rehousing may reduce the effect of cagemate number on mortality. To test this hypothesis a study and intervention could be designed where cage density is intentionally manipulated at age of life points—with or without replacement of cagemates.

In conclusion, our study highlights the importance of housing density, specifically the presence of cagemates, in relation to hazard of death and overall survival outcomes in mice. We have analyzed the influence of changes in cage density over time on death rates in male and female mice, offering insights into the differential associations of social contexts on survival outcomes. Our findings indicate sex-specific responses to variations in cage density underscoring the importance of social conditions in influencing survival in laboratory environments using both male and female mice. These findings may aid in the improvement of housing protocols and experimental designs to enhance the welfare of laboratory mice and validity of investigations in mice, as social dynamics influence their health and longevity. At a minimum, consideration should be given to study design, analyses, and interpretation for the potential associations with outcomes related to ‘survivor bias’, maximum longevity observations, and other aging-related or survivor-associated endpoints. Subsequent research may elucidate the fundamental mechanisms governing these relationships, providing significant insights for both laboratory studies and animal welfare.

## Data Availability

We used publicly available datasets generated by Harrison, D.E., et al. The raw data analyzed are publicly accessible through the Mouse Phenome Database (https://phenome.jax.org/projects/ITP1), maintained by The Jackson Laboratory, Bar Harbor, ME.

## Code availability

Analytical code used in this study are available in the Open Science Framework (OSF) repository (https://osf.io/5q3tm/; https://doi.org/10.17605/OSF.IO/5Q3TM).
